# Supplementary material for: Identification of Epigenetic Interactions between MicroRNA-30c-5p and DNA Methyltransferases in Neuropathic Pain
Source: Int J Mol Sci. 2022 Nov 13;23(22):13994. doi: 10.3390/ijms232213994 (PMC9694031; doi:10.3390/ijms232213994)
Supplement: Supplementary file 1 [file ijms-23-13994-s001.zip › ijms-1973173-supplementary.pdf]

Figure S1, S2, S3 and S4 are detailed in this section.

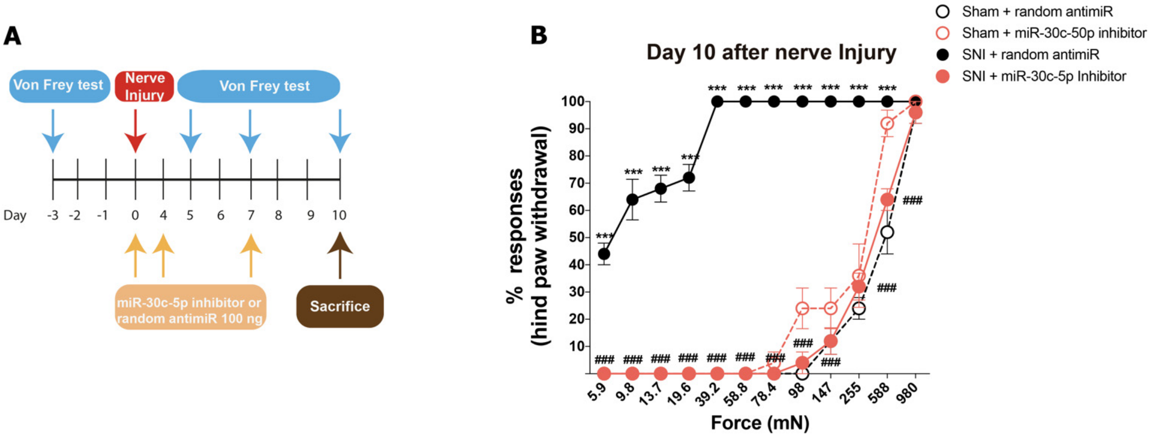

Figure S1. miR-30c-5p inhibitor prevents neuropathic pain after sciatic nerve injury in rats. A: Intracisternal administration protocol of miR-30c-5p inhibitor or random anti-miR. Rats subjected to SNI received a cycle of three intracisternal injections of miR-30c-5p-inhibitor (100 ng/10µl; n = 5) or random anti-miR sequence (n = 5). The first administration was at the time of SNI, and two more injections were administered on days 4 and 7 after SNI surgery. Sham rats treated with random anti-miR (n = 5) served as controls. B: The curves represent the percentage of hind paw withdrawal responses induced by von Frey monofilaments of increasing force (mN) in sham and SNI rats treated with miR-30c-5p-inhibitor or random anti-miR, 10 days after SNI or sham surgery. ***p<0.001 vs sham + random anti-miR; ###p<0,001 vs 10-days SNI + random anti-miR (mixed-design three-way ANOVA followed by Bonferroni post hoc test).

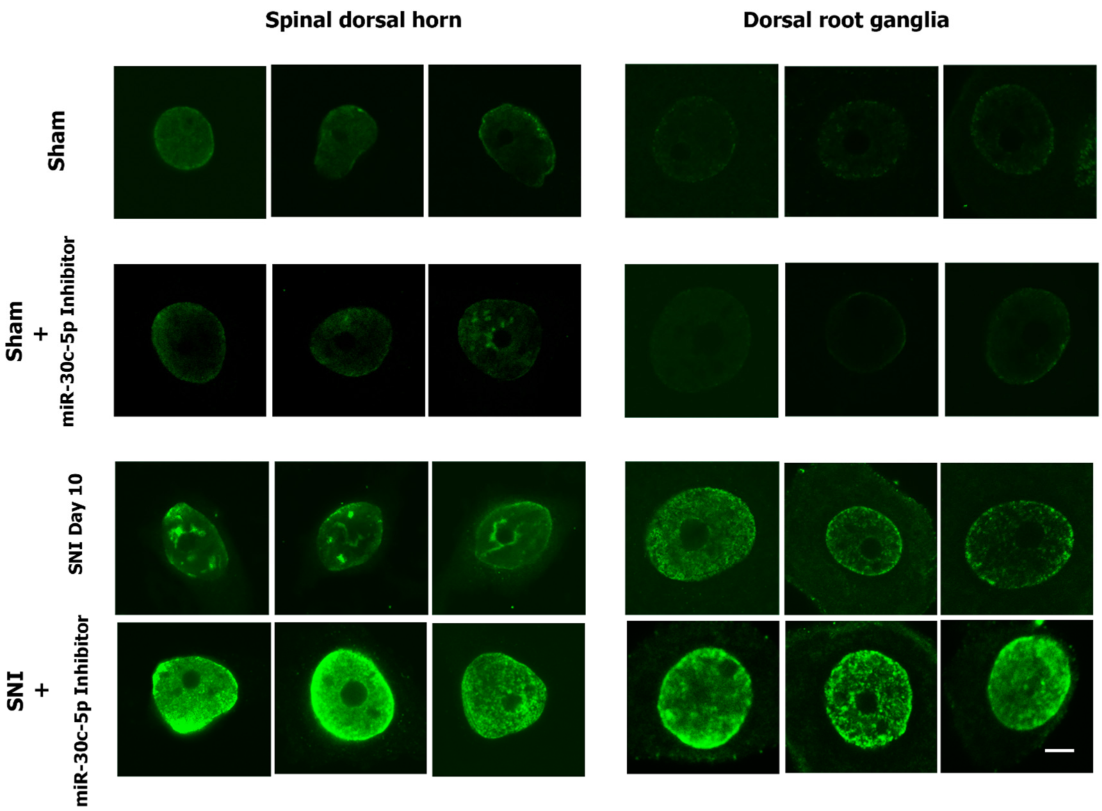

Figure S2. Images showing 5′-methylcytosine (5′-MeC) positive immunostaining in neurons isolated from the spinal dorsal horn and dorsal root ganglia from sham rats treated with random anti-miR; sham rats treated with miR-30c-5p-inhibitor; 10 days-SNI rats treated with random anti-miR; and 10 days-SNI rats treated with miR-30c-5p-inhibitor. Scale bar: 5 µm.

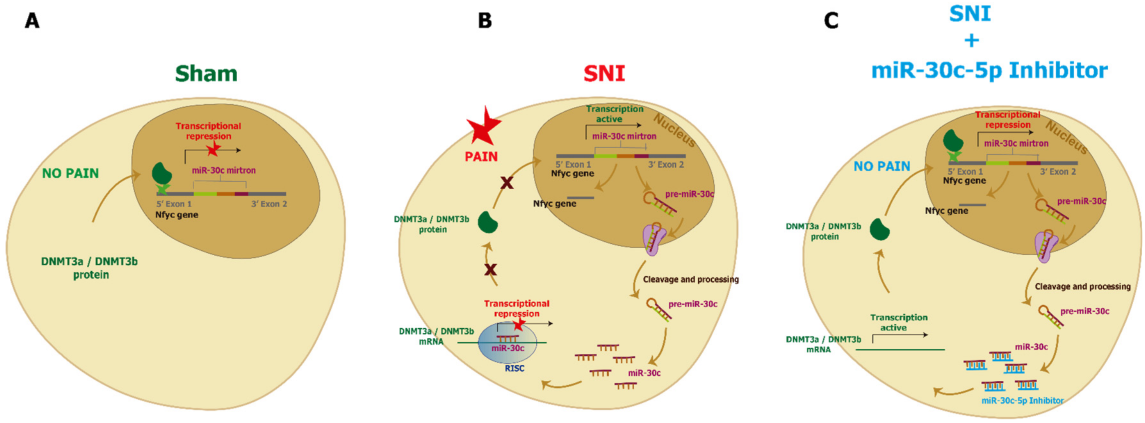

Figure S3. Schematic representation of miR-30c-5p and Nfyc modulatory feedback. In non-pain conditions (Sham rats) (**A**), DNMT3a/DNMT3b protein methylates Nfyc promoter, leading to transcriptional repression of Nfyc and therefore of miR-30c mirtron. In pain conditions after sciatic nerve injury (SNI) (**B**) the levels of miR-30c-5p significantly increases in pain-related areas such as the SDH and DRG. MiR-30c-5p interacts then with the 3′ UTR of its targets DNMT3a/DNMT3b, inducing the mRNA degradation and translational repression. The promoter region of Nfyc will be, then, no longer methylated allowing the synthesis of more miR-30c-5p and contributing to the pain state. In no pain conditions induced by the administration of miR-30c-5p inhibitor after SNI (**C**), the increased levels of miR30c-5p will be neutralized, leading to a general loss of activity of miR-30c-5p. Since miR-30c-5p is no longer active, transcription and translation of DNMT3a/DNMT3b can occur, leading to a

hypermethylation state of Nfyc promoter and therefore to transcriptional repression of miR-30c-5p which may contribute to the long-lasting antiallodynic effect produced by treatment with miR-30c-5p inhibitor in SNI rats.

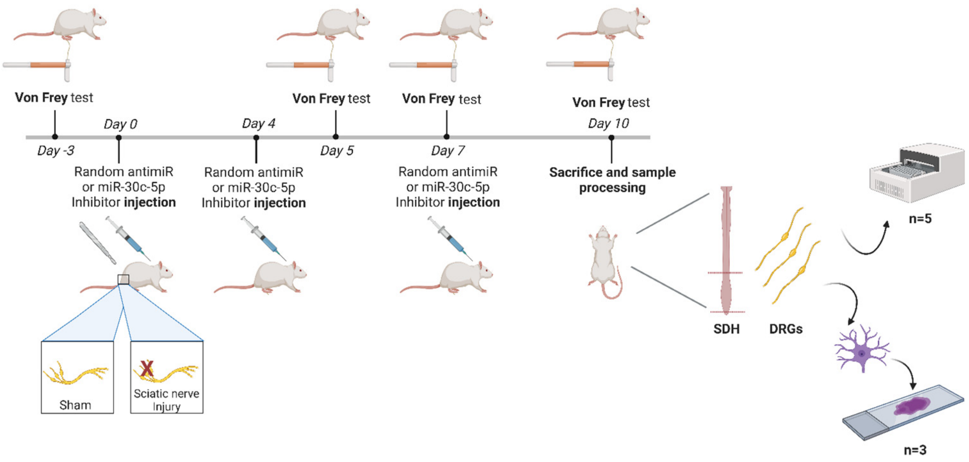

Figure S4. Study design. We assessed the role and influence of miR-30c-5p in the aberrant DNA methylation that follows peripheral nerve injury. We performed studies in rats subjected to SNI and in cultured cells. Rats were subjected to SNI and injected with a miR-30c-5p inhibitor or a random anti-miR to identify via immunofluorescence studies, qPCR, and western blot, the influence of miR-30c-5p inhibition on global DNA methylation levels and the relative expression of the two main DNMTs (DNMT3a and DNMT3b) in the SDH and DRGs.
